# Correction: Inhibitors of lysosomal function or serum starvation in control or LAMP2 deficient cells do not modify the cellular levels of Parkinson disease-associated DJ-1/PARK 7 protein

**DOI:** 10.1371/journal.pone.0233091

**Published:** 2020-05-07

**Authors:** Raúl Sánchez-Lanzas, José G. Castaño

There is an error in Fig 7. It is not clearly indicated that lanes 4 and 5 of the western blot images are non-adjacent lanes taken from the same blot. A corrected Fig 7 is provided here.

**Fig 7:**
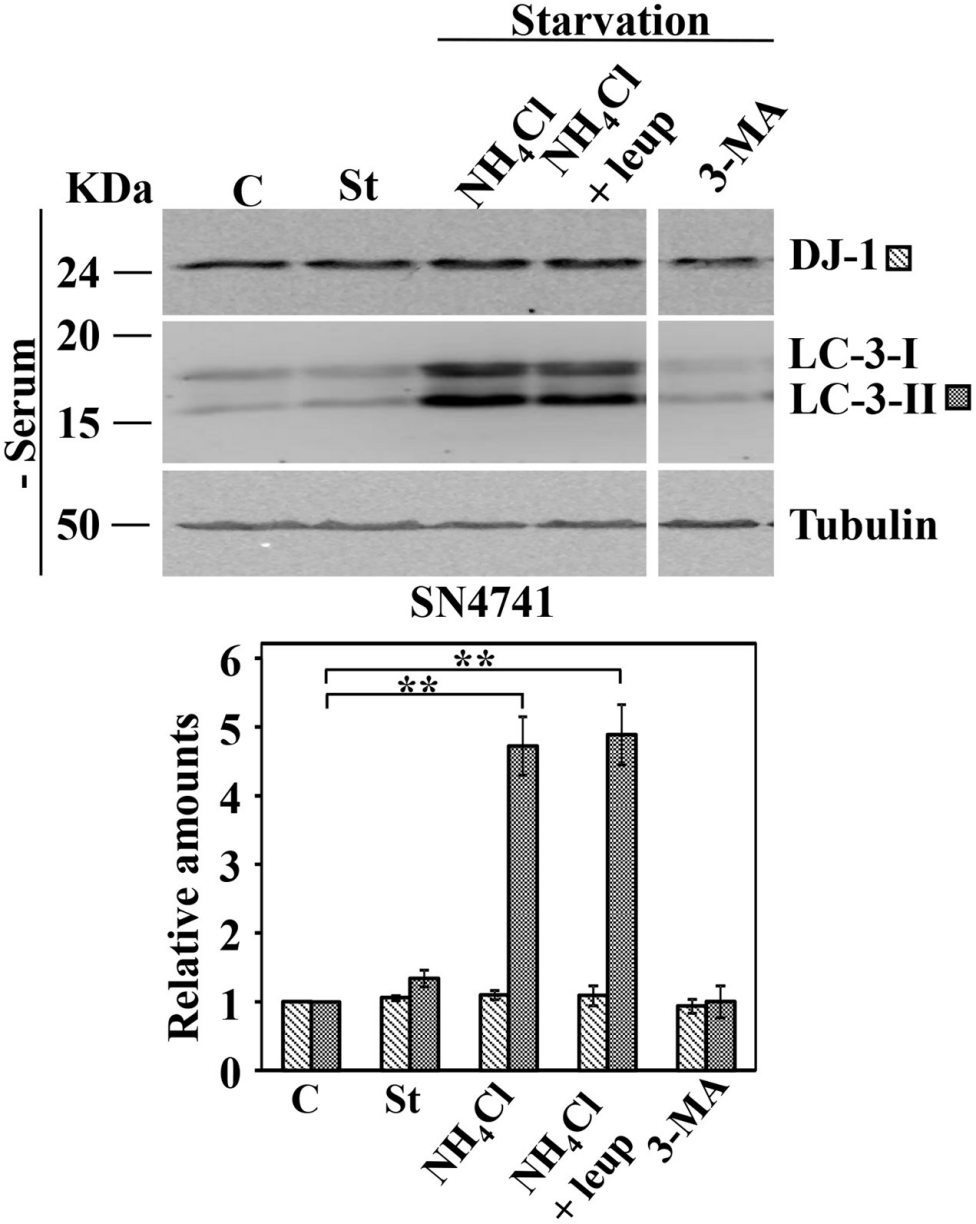
Protein expression levels of DJ-1 following activation of CMA by serum starvation in SN4741 cells. Exponentially growing SN4741 cells were kept in complete medium (C) or starved of serum for 24 h in the absence (St) or in the presence of NH_4_Cl, NH_4_Cl and leupeptin (Leup), or 3-methyl adenine (3-MA). Panels show the effect of serum deprivation in SN4741. Total cell lysates were analysed by Western and immunoblot with the corresponding specific antibodies, as indicated. Anti-tubulin antibodies were used as total protein loading control. Graphs below each panel show the quantification of the levels of the different proteins analysed respect to the levels in cells kept in complete growth medium, controls. Values are expressed as mean ± s.e.m. from three different experiments. Significant differences between groups ** at p<0.01 by Student t-test are indicated.

Please see the Supporting Information files below for the underlying blots for Fig 7, as well as the underlying datasets for all charts presented in the article.

The authors indicate that underlying images for other figures are available upon request.

## Supporting information

S1 File Uncropped blots underlying Fig 7. (ZIP)

S2 File Data underlying charts. (ZIP)

## References

[1] Sánchez-Lanzas R, Castaño JG (2018) Inhibitors of lysosomal function or serum starvation in control or LAMP2 deficient cells do not modify the cellular levels of Parkinson disease-associated DJ-1/PARK 7 protein. PLoS ONE 13(7): e0201152 10.1371/journal.pone.0201152 30048497 PMC6062081

