# Supplementary figures and images for: Inhibitors of lysosomal function or serum starvation in control or LAMP2 deficient cells do not modify the cellular levels of Parkinson disease-associated DJ-1/PARK 7 protein
Source: PLoS One. 2018 Jul 26;13(7):e0201152. doi: 10.1371/journal.pone.0201152 (PMC6062081; doi:10.1371/journal.pone.0201152)

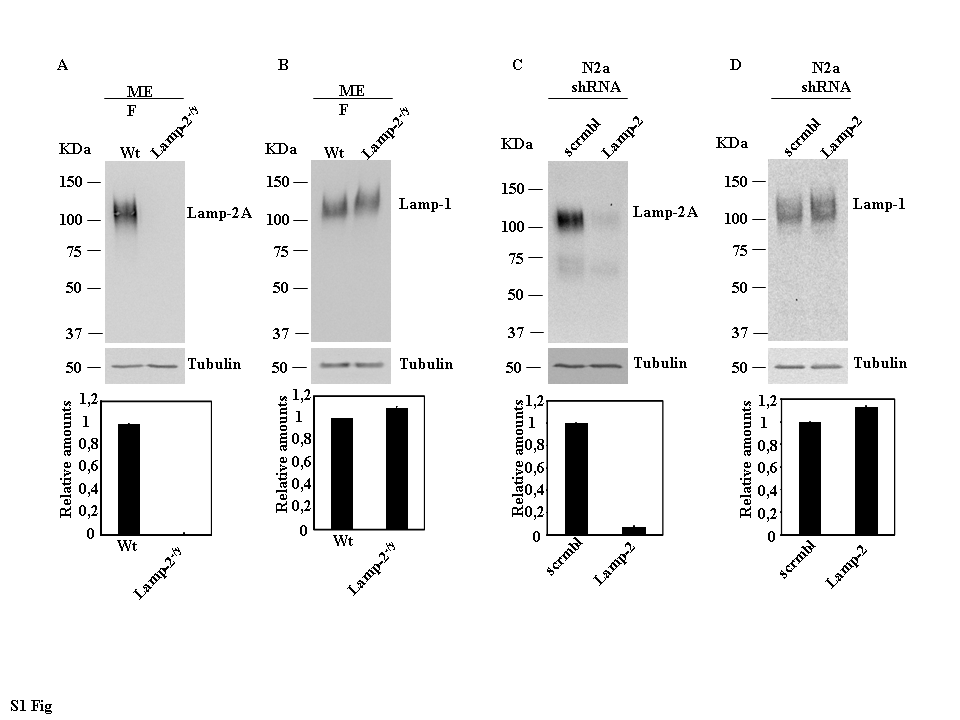

Supplement: S1 Fig — Total lysates from exponentially growing control and Lamp2-deficient MEF (A, B) and N2a (C, D) cells were prepared and the expression levels of Lamp-2A (A, C) and Lamp-1 (B, D) membrane lysosomal proteins were analysed by Western and immunoblot with the corresponding specific antibodies, as indicated. Anti-tubulin antibodies were used as protein loading control. Below each panel is shown the graph of quantification of the corresponding immunoblots. Data are mean ± s. e. m. from at least three different experiments. (TIF) [file pone.0201152.s001.TIF]

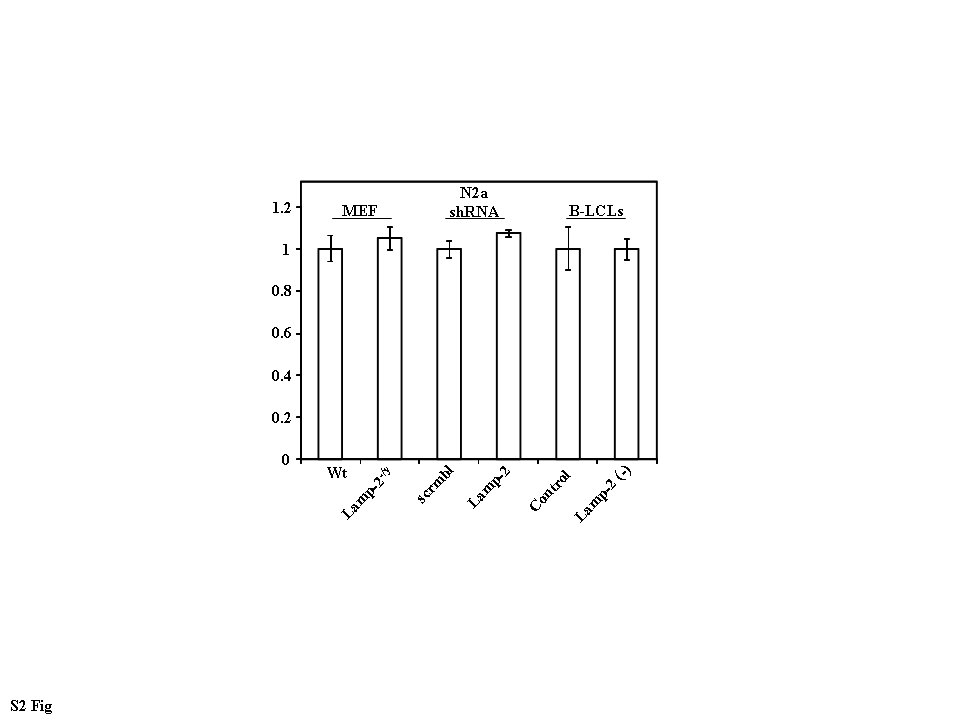

Supplement: S2 Fig — Exponentially growing control and Lamp-2-deficient MEF, N2a, B-LCLs cells were cultured in complete medium and total RNA was isolated and analyzed by qRT-PCR, as described under "Materials and methods". Graph shows the relative fold change using β-actin mRNA levels, as reference. Data are average from two experiments assayed by triplicate (technical replica), upper and lower values are represented by horizontal lines. (TIF) [file pone.0201152.s002.TIF]

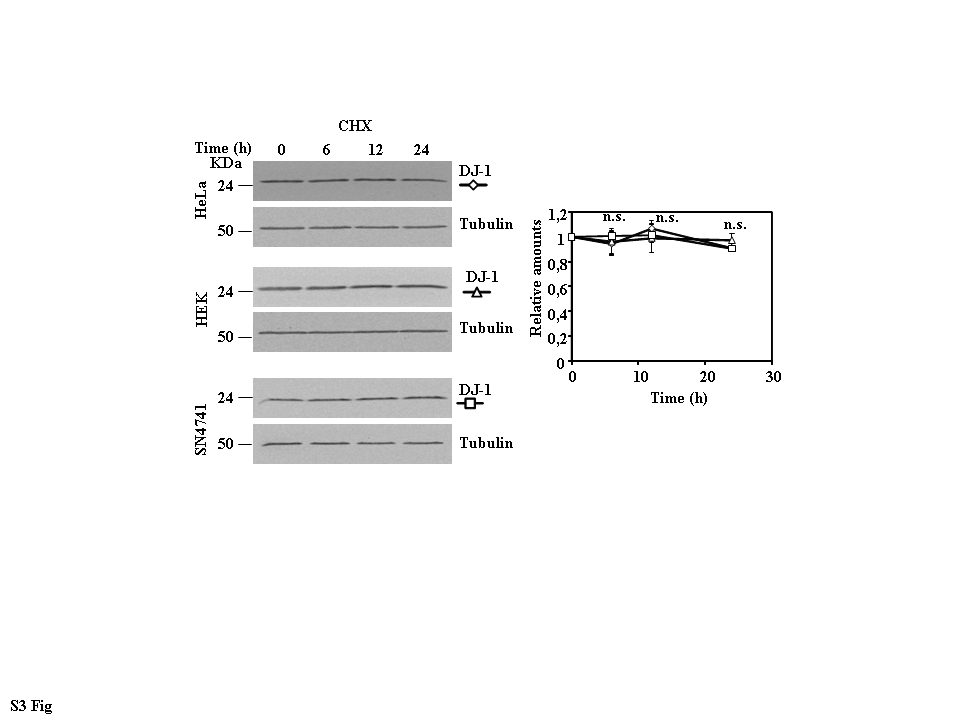

Supplement: S3 Fig — Exponentially growing HeLa (A), HEK (B) and SN4741 (C) cells were treated with cycloheximide (CHX) for the times indicated. Total cell lysates were prepared and DJ-1 protein levels were analyzed by Western and immunoblot with specific antibodies. Anti-tubulin antibodies were used as total protein loading control. Right graph shows the quantification of the levels of DJ-1 protein. Values are expressed as mean ± s.e.m. from three different experiments. (TIF) [file pone.0201152.s003.TIF]

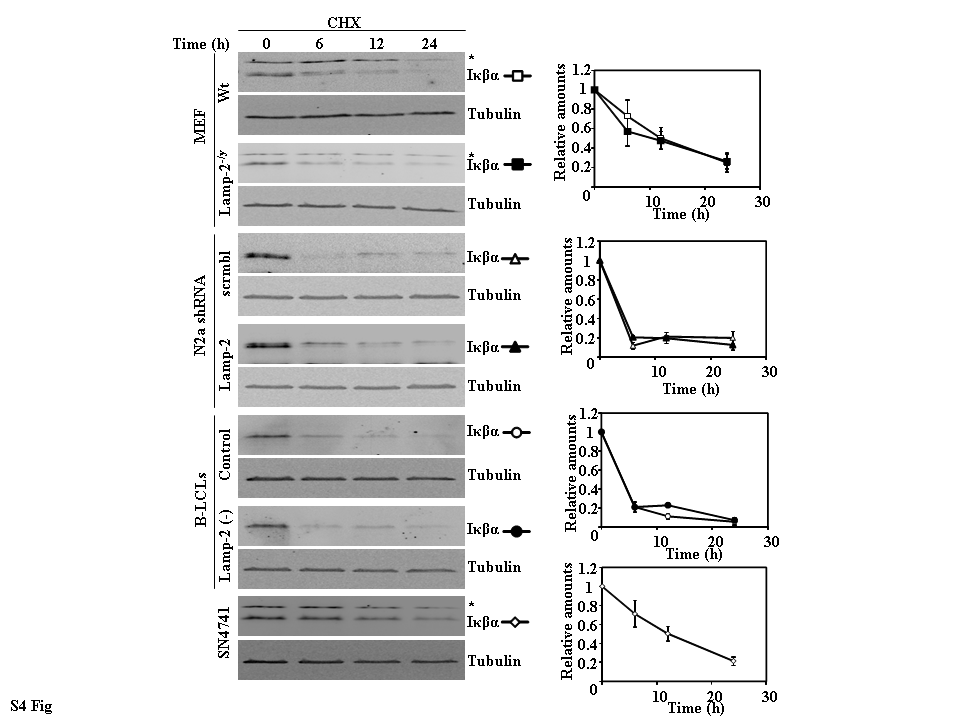

Supplement: S4 Fig — Exponentially growing cells from control and Lamp-2-deficient cells were treated with cycloheximide (CHX) for the times indicated. Total cell lysates were prepared and IKappaBα (Iκbα) protein levels were analysed by Western and immunoblot with specific antibodies. Anti-tubulin antibodies were used as total protein loading control. Panels show the results obtained with MEF, N2a, and B-LCLs and SN4741 cell lines, Graphs on the right side show the quantification of the levels of IKappaBα protein respect to their corresponding untreated cells as controls (time 0 h). Values are expressed as mean ± s.e.m. from three different experiments, no significant differences in degradation was found. (TIF) [file pone.0201152.s004.TIF]

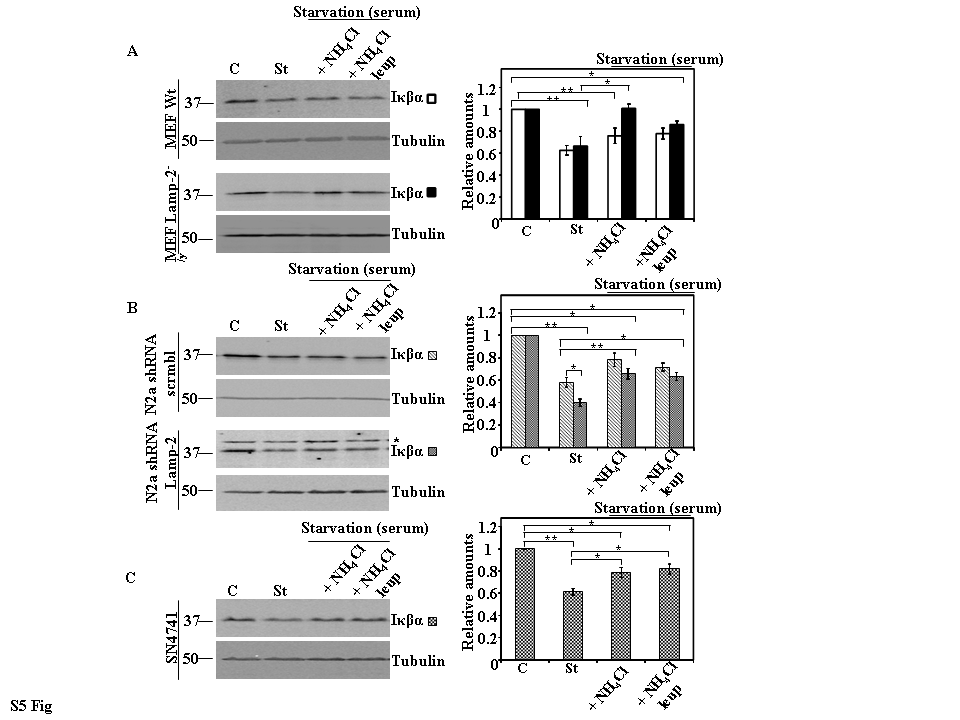

Supplement: S5 Fig — Exponentially growing control and Lamp-2-deficient cells and SN4741 were kept in complete medium (C) or starved of serum for 24 h in the absence (St) or in the presence of NH4Cl or NH4Cl and leupeptin (leup). Panel A shows the results obtained in MEF Wt cells and Lamp-2-deficient (Lamp-2-/y) cells. Panel B shows the results obtaine from N2a shRNA scrmbl cells and Lamp-2-deficient N2a shRNA Lamp-2 cells. Panel C shows the resutls obtained with SN4741 Total cell lysates were analysed by Western and immunoblot with the corresponding specific antibodies: as indicated. Anti-tubulin antibodies were used as total protein loading control. Graphs show the quantification of the levels of the different proteins analysed respect to the levels in cells kept in complete growth medium, controls. Values are expressed as mean ± s.e.m. from three different experiments. Significant differences between the indicated pairs analyzed by Student t-test are indicated by ** at p<0.01. and * p<0.05. (TIF) [file pone.0201152.s005.TIF]
