# Supplementary figures and images for: Correction: Inhibitors of lysosomal function or serum starvation in control or LAMP2 deficient cells do not modify the cellular levels of Parkinson disease-associated DJ-1/PARK 7 protein
Source: PLoS One. 2020 May 7;15(5):e0233091. doi: 10.1371/journal.pone.0233091 (PMC7205197; doi:10.1371/journal.pone.0233091)

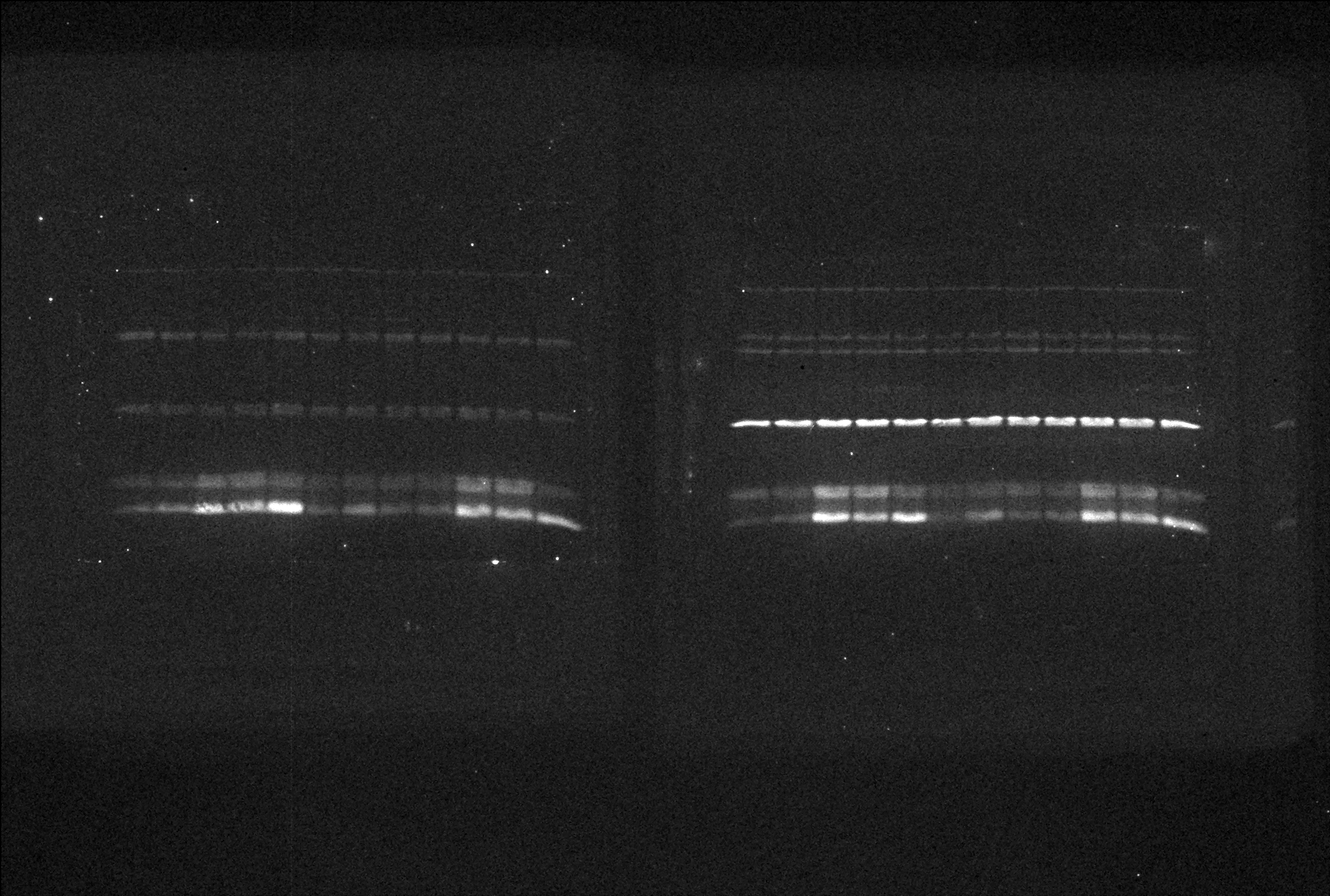

Supplement: S1 File — (ZIP) [file pone.0233091.s001.zip › S1 File/DJ-1.tif]

-Serum

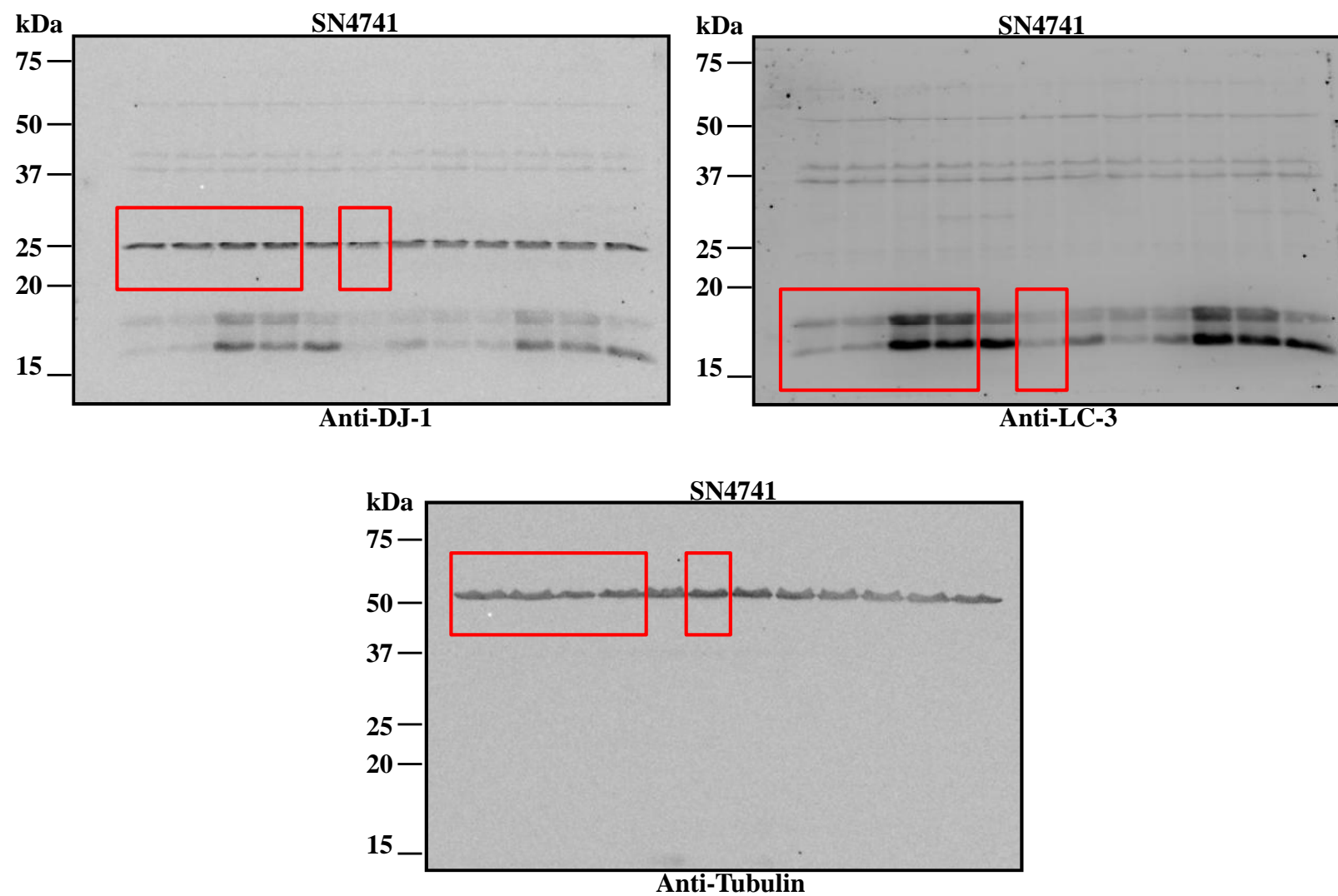

Figure 7

Supplement: S1 File — (ZIP) [file pone.0233091.s001.zip › S1 File/Figure 7 uncropped blots.pdf]

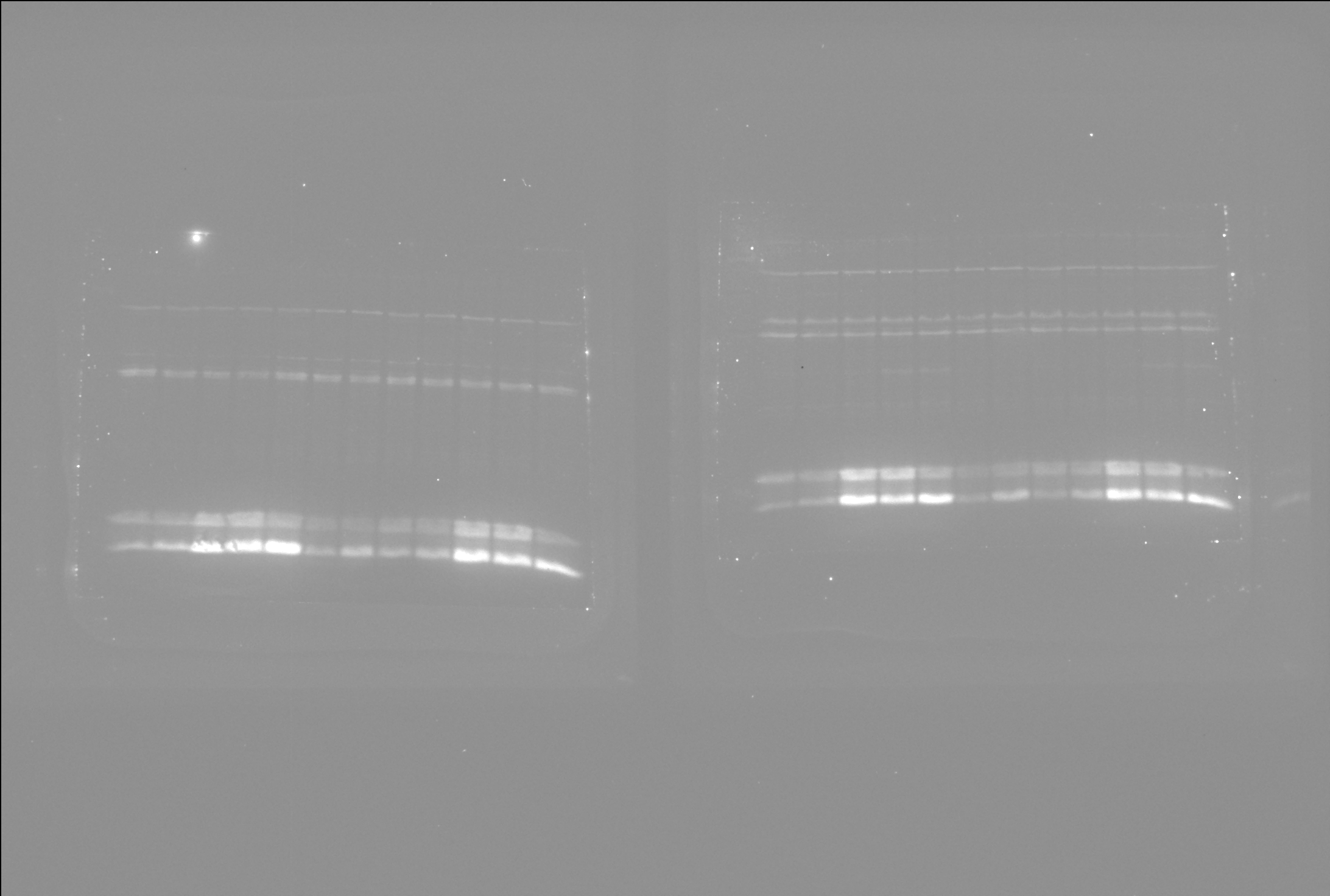

Supplement: S1 File — (ZIP) [file pone.0233091.s001.zip › S1 File/LC-3.tif]

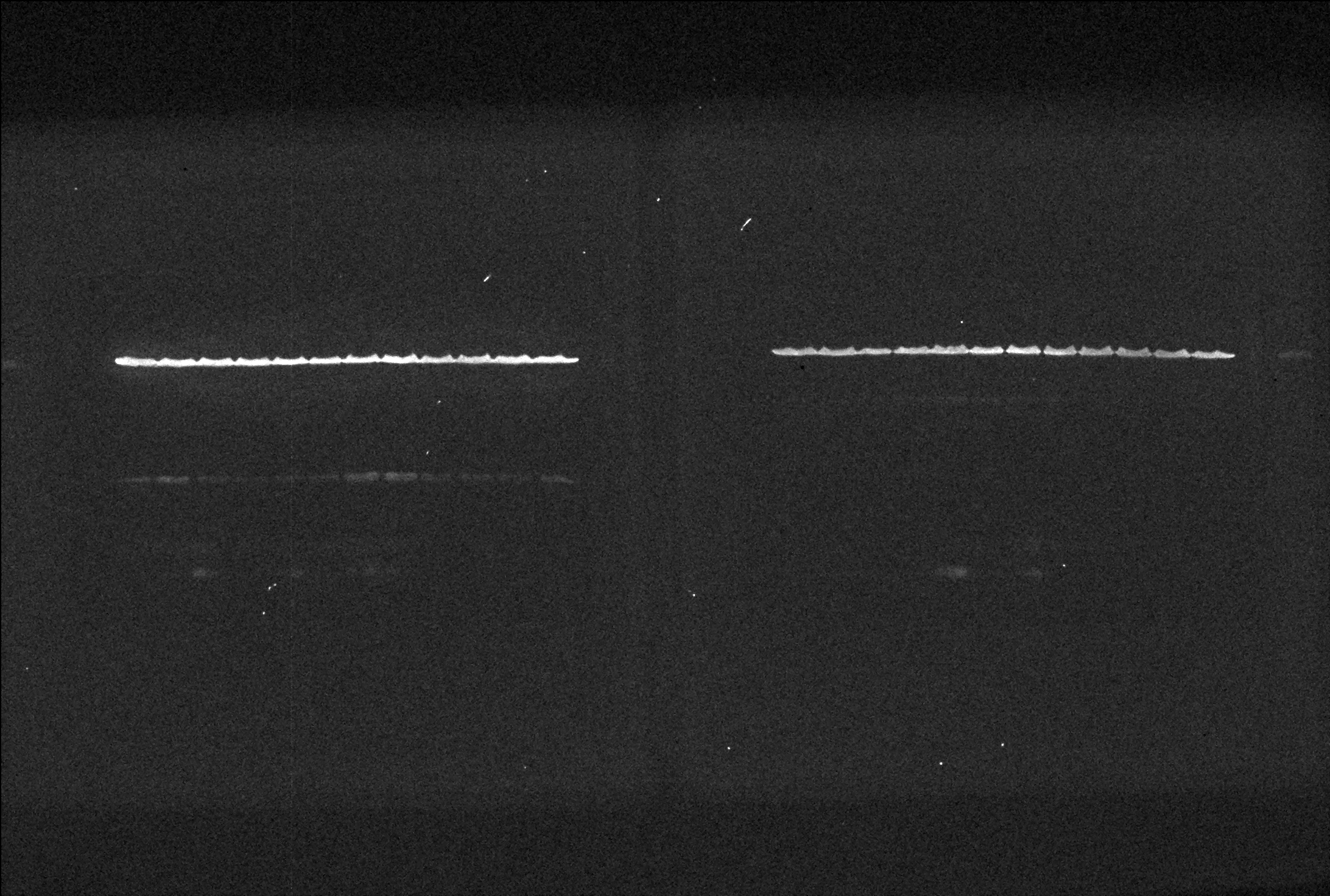

Supplement: S1 File — (ZIP) [file pone.0233091.s001.zip › S1 File/Tubulin.tif]
