# Supplementary material for: Correction: Inhibitors of lysosomal function or serum starvation in control or LAMP2 deficient cells do not modify the cellular levels of Parkinson disease-associated DJ-1/PARK 7 protein
Source: PLoS One. 2020 May 7;15(5):e0233091. doi: 10.1371/journal.pone.0233091 (PMC7205197; doi:10.1371/journal.pone.0233091)
Supplement: S1 File — (ZIP) [file pone.0233091.s001.zip › S1 File/Visualization instructions. Read this text to visualize and interpret original blots for Figure 7.doc]

In order to properly open and visualize the original files containing the Western blot images with Adobe Photoshop, the following instructions should be considered:

- Once the desired image file has been opened, the Rectangular Marquee Tool has to be selected and a wide selection area (almost including the edges of the image but without touching them) should be drawn (see below).

- Next, on the “Image” panel, the Auto Tone command should be selected. At this point, the different bands should be visualized (see below).

- Finally, the Invert command should be selected (Image-Adjustments-Invert). In order to better visualize the bands the parameters brightness and contrast can be adjusted.

The following consideration should be taken into account for the proper identification of the blot images that have been used in the figure 7 panels:

**Figure 7**: In the original blot image corresponding to SN4741 there are two membranes, the one on the right side has been used for DJ-1, LC-3 and tubulin antibodies.
